# Predictors and dynamic online nomogram for postoperative delayed hyponatremia after endoscopic transsphenoidal surgery for pituitary adenomas: a single-center, retrospective, observational cohort study with external validation

**DOI:** 10.1186/s41016-023-00334-3

**Published:** 2023-08-01

**Authors:** Xiangming Cai, An Zhang, Peng Zhao, Zhiyuan Liu, Yiliyaer Aili, Xinrui Zeng, Yuanming Geng, Chaonan Du, Feng Yuan, Junhao Zhu, Jin Yang, Chao Tang, Zixiang Cong, Yuxiu Liu, Chiyuan Ma

**Affiliations:** 1 Department of Neurosurgery, Jinling Hospital, Nanjing, China; 2 School of Medicine, Southeast University, Nanjing, China; 3 Department of Molecular Cell Biology and Immunology, Amsterdam UMC, Vrije Universiteit Amsterdam, Amsterdam, Netherlands; 4 Department of Neurosurgery, The First Affiliated Hospital of Nanjing Medical University, Nanjing, China; 5 Department of Neurosurgery, The Affiliated Jinling Hospital of Nanjing Medical University, Nanjing, China; 6 Department of Neurosurgery, Affiliated Jinling Hospital, Medical School of Nanjing University, Nanjing, China; 7 Department of Critical Care Medicine, Jinling Hospital, Nanjing Medical University, Nanjing, China; 8 Department of Biostatistics, School of Public Health, Southern Medical University, Guangzhou, China; 9 Department of Neurosurgery, Jinling Hospital, the First School of Clinical Medicine, Southern Medical University, Nanjing, China

**Keywords:** Endoscopic transsphenoidal surgery, Nomogram, Pituitary adenomas, Postoperative delayed hyponatremia, Predictors

## Abstract

**Background:**

Postoperative delayed hyponatremia (PDH) is a major cause of readmission after endoscopic transsphenoidal surgery (eTSS) for pituitary adenomas (PAs). However, the risk factors associated with PDH have not been well established, and the development of a dynamic online nomogram for predicting PDH is yet to be realized. We aimed to investigate the predictive factors for PDH and construct a dynamic online nomogram to aid in its prediction.

**Methods:**

We analyzed the data of 226 consecutive patients who underwent eTSS for PAs at the Department of Neurosurgery in Jinling Hospital between January 2018 and October 2020. An additional 97 external patients were included for external validation. PDH was defined as a serum sodium level below 137 mmol/L, occurring on the third postoperative day (POD) or later.

**Results:**

Hyponatremia on POD 1–2 (OR = 2.64, *P* = 0.033), prothrombin time (PT) (OR = 1.78, *P* = 0.008), and percentage of monocytes (OR = 1.22, *P* = 0.047) were identified as predictive factors for PDH via multivariable logistic regression analysis. Based on these predictors, a nomogram was constructed with great discrimination in internal validation (adjusted AUC: 0.613–0.688) and external validation (AUC: 0.594–0.617). Furthermore, the nomogram demonstrated good performance in calibration plot, Brier Score, and decision curve analysis. Subgroup analysis revealed robust predictive performance in patients with various clinical subtypes and mild to moderate PDH.

**Conclusions:**

Preoperative PT and the percentage of monocytes were, for the first time, identified as predictive factors for PDH. The dynamic nomogram proved to be a valuable tool for predicting PDH after eTSS for PAs and demonstrated good generalizability. Patients could benefit from early identification of PDH and optimized treatment decisions.

**Supplementary Information:**

The online version contains supplementary material available at 10.1186/s41016-023-00334-3.

## Background

Management of pituitary adenomas (PAs) includes surgical resection, medications, and radiotherapy. Endoscopic transsphenoidal surgery (eTSS) is a common treatment approach for PAs. Postoperative delayed hyponatremia (PDH) occurs in 1.8–35% of patients who undergo eTSS [1]. Patients with PDH may experience headaches, nausea, coma, and even death [2]. Additionally, readmission due to PDH [3] has restricted the development of day-care units for PAs.

Previous research has reported various predictors for PDH, including old age, serum sodium concentrations on postoperative days (POD) 1 and 2 [2–5], postoperative diabetes insipidus, diaphragma sellae sinking depth, and postoperative length of the “measurable pituitary stalk” [6]. However, the relation between tumor type, tumor size, and PDH has not reached a consistent conclusion [1]. Also, there is currently no dynamic online clinical model available for predicting the occurrence of PDH after eTSS for PAs. Nomogram is a user-friendly integrated predictive tool, providing visualization of complex predictive models by incorporating multiple predictors [7]. Lin et al. developed a nomogram to predict PDH [6]. However, the potential predictors included in their study were not thoroughly explored, and the nomogram they constructed was not developed as an online tool, which restricted its practical application.

In this study, we aimed to thoroughly explore the predictive factors for PDH and further establish the first predictive dynamic online nomogram forecasting PDH after eTSS for PAs.

## Methods

### Patient selection and data collection

This retrospective study included patients who underwent eTSS for PAs between January 2018 and October 2020 at the Neurosurgery Department of Jinling Hospital. Additionally, a total of 97 PA patients from The First Affiliated Hospital of Nanjing Medical University were included for external validation. The criteria for inclusion were as follows: (1) pathologically confirmed PA, (2) patients who underwent eTSS, (3) patients with laboratory assessment of serum sodium concentration on the POD 3 or later (outcome variable), and (4) patients with at least one collected variable. The exclusion criteria included: (1) PAs not confirmed by pathology, (2) patients without the outcome variable or patients who did not have any collected variables, and (3) patients with hyponatremia lasting from POD 1 and 2 to POD 3 or later.

Our institutional research ethics board approved this retrospective study (2021NZKY-037–01) and waived the requirement for obtaining informed consent due to the retrospective nature of the research and data anonymization.

Hyponatremia was defined as serum sodium concentration < 137 mmol/L based on the normal range of serum sodium concentration in our institution. There are many incidence time points for PDH in the literature. We defined PDH as hyponatremia occurring on POD 3 and onwards in the current study, as most studies included in Lee et al.’s meta-analysis applied this criteria [1]. The collected clinical characteristics included age, gender, treatment history of PAs, preoperative signs and symptoms (acromegaly, moon face, visual impairment, visual field defect, and headache), primary-recurrence subtype, and clinical subtypes (nonfunctioning, prolactin (PRL) secreting [8], growth hormone (GH) secreting [8, 9], and adrenocorticotropic hormone (ACTH) secreting [10] PAs). The following radiological features were also collected: Hardy grade, Knosp grade, tumor size (lengths of tumor maximum dimension, height, width, and thickness), tumor shape 1 (in sella, ellipsoid, or hourglass signs), tumor shape 2 (lobulated shape), sellar barrier (weak or strong) [11], optic nerve compression, pituitary apoplexy, and tumor signal intensity (T2-weighted magnetic resonance imaging (MRI) signal intensity compared with that of the white matter). For Hardy grade for sellar invasion [12] and Knosp grade [13] were used to assess invasion. Grades 0–2 were classified as the noninvasive while grades 3–4 were categorized as invasive. Residual tumor was evaluated as a binary variable from postoperative magnetic resonance imaging (MRI). The following preoperative serum laboratory assessments were extracted: pituitary hormones, complete blood count, renal and hepatic functions, coagulation profile, and electrolytes levels. Based on the laboratory cutoffs in our institution, the normal range of prothrombin time (PT) was from 9 to 14 seconds, and that of the percentage of monocytes was from 3 to 10%. Hyponatremia before surgery and on POD 1–2 were extracted as binary variables. We also collected polyuria on POD 1–2 as a binary variable, defined as urine output exceeding 4000 ml/day [14].

### Perioperative management

Patients will be transferred to intensive care unit (ICU) after operation. On POD 2, if their condition is stable, they will be transferred to general ward for further care. Input and output of patients are routinely recorded in ICU, and urine output will be monitored until discharge. In general, electrolytes levels, including serum sodium concentration, are monitored within 24 hours after operation and on POD 3 and 5 as well. If any abnormal items were detected, more intensive monitoring of electrolytes levels will be applied. For patients with mild hyponatremia, adjusted diet with more sodium salt will be recommended. In case of moderate to severe hyponatremia or corresponding symptoms, including nausea, vomiting, and changes in mental state, patients will be admitted with oral sodium supplementation or sodium intravenous infusion. Desmopressin will be used to treat patients with polyuria.

### Statistical analyses and development of the nomogram

Model construction processes were carried out based on the “Transparent Reporting of a Multivariable Prediction Model for Individual Prognosis or Diagnosis” (TRIPOD) guidance (Supplementary Table 1) [15]. Variables with more than 25% missing data were excluded from the analysis. The Iterative Markov chain Monte Carlo method was used to impute missing values until convergence with the “mice” R package (version 3.13.0) [16]. Five imputed datasets were constructed (*N* = 226) (Supplementary Fig. 2). Uni- and multi-variable logistic regression analyses were performed separately on each imputed dataset. And the estimates from these analyses were then pooled together using Rubin’s rules [17] with the “mice” R package.

Continuous data were described as the mean ± standard deviation (SD). Because logistic regression analysis was applied in the current study, continuous variables without normal distributions were either transformed into the logarithmic scale or recoded as binary variables. Interaction between collected variables were explored; however, no clinical meaningful interactions were detected. First, a comparison of variables was performed between groups with and without PDH. Student’s *t* test was used to compare two continuous variables, while the chi-squared test or Fisher’s exact test was applied for categorical variables. Then, univariable logistic regression analysis was conducted for all variables. Variables were filtered based on statistical significance in the comparison between the groups with and without PDH, as well as the results of the univariable logistic regression analysis. Collinearity among these filtered variables was assessed with Spearman correlation analysis. Variables demonstrating collinearity were selectively removed based on their clinical significance. Finally, multivariable regression analysis was conducted to identify the independent predictors for PDH.

Complete dataset (*N* = 156) was extracted and used to develop nomogram models with these 5 imputed datasets, respectively. Independent predictors were integrated and visualized as a nomogram model using the “rms” R package (version 6.1.0). The events per variable (EVP) = 10 criteria was used as the sample size estimation method for the current risk prediction model [7, 18]. The receiver operating characteristic (ROC) curve was computed to evaluate the model’s discrimination with “pROC” (version 1.17.0.1). Internal validation methods, including cross-validation, jackknife validation, and bootstrap validation were applied to compute the adjusted area under curve (AUC) values for the model. Calibration of the model was assessed with the calibration curve implemented in the “rms” R package. The overall performance of the nomogram model was qualified as the scaled Brier Score, which ranges from 0.0 (perfect) to 0.25 (worthless) [19]. Decision curve analysis (DCA) was performed to evaluate the clinical benefit of the model with the “DecisionCurve” R package (version 1.3). We further evaluated the performance of the nomogram model in the following subgroups: clinical subtypes, menstruation status, and PDH severity subgroups. An interactive web-based dynamic nomogram application was constructed with the “DynNom” R package (version 5.0.1) and Shiny website (www.shinyapps.io). The R software (version 3.6.0) was used for statistical analyses in the current research, and *P* < 0.05 and 0.1 were considered statistically significant and marginally statistically significant, respectively.

## Results

### Characteristics of patients and variables

A total of 226 patients were eligible for inclusion in the current research and were used as the training dataset. Ninety-seven patients were included as external validation dataset. Missing data patterns and percentages for the training dataset are displayed in Supplementary Fig. 1 and Supplementary Table 2. The overall characteristics of the training dataset are shown in Table 1 and Supplementary Table 3. Out of the 226 patients in training dataset, 63 (27.9%) developed PDH. Additionally, the external validation dataset had 17 patients with PDH.

**Table 1:** Partial important characteristics of patients in the without PDH group and in the with PDH group

Variables	Without PDH	With PDH	*P*
	(*N* = 163)	(*N* = 63)	
Age (year)	49.91 ± 13.11	52.78 ± 13.59	0.153
Gender			0.755
Female	85 (52.1%)	35 (55.6%)	
Male	78 (47.9%)	28 (44.4%)	
Clinical subtype^a^			0.650
Nonfunctioning	107 (67.3%)	41 (66.1%)	
PRL secreting	7 (4.4%)	6 (9.7%)	
GH secreting	35 (22.0%)	12 (19.4%)	
ACTH secreting	7 (4.4%)	2 (3.2%)	
PRL-GH secreting	3 (1.9%)	1 (1.6%)	
Preoperative hyponatremia^b^			1.000
No	151 (95.6%)	57 (95.0%)	
Yes	7 (4.4%)	3 (5.0%)	
Hyponatremia on POD 1–2^c^			0.098^*^
No	139 (90.8%)	48 (82.8%)	
Yes	14 (9.2%)	10 (17.2%)	
Polyuria			0.609
No	131 (80.4%)	48 (76.2%)	
Yes	32 (19.6%)	15 (23.8%)	
Visual impairment^d^			0.040^**^
No	83 (51.2%)	22 (34.9%)	
Yes	79 (48.8%)	41 (65.1%)	
Hardy grade for suprasellar extension^b^			0.038^**^
0	38 (24.2%)	15 (24.6%)	
A	41 (26.1%)	7 (11.5%)	
B	46 (29.3%)	16 (26.2%)	
C	28 (17.8%)	18 (29.5%)	
D	2 (1.3%)	2 (3.3%)	
E	2 (1.3%)	3 (4.9%)	
Hardy grade for sellar invasion^b^			0.264
Noninvasive	119 (75.8%)	41 (67.2%)	
Invasive	38 (24.2%)	20 (32.8%)	
Knosp grade^e^			0.220
Noninvasive	99 (62.7%)	32 (52.5%)	
Invasive	59 (37.3%)	29 (47.5%)	
Tumor shape 1^f^			0.067^*^
In sella	21 (16.2%)	9 (18.8%)	
Ellipsoid	51 (39.2%)	10 (20.8%)	
Hourglass sign	58 (44.6%)	29 (60.4%)	
Tumor shape 2^f^			0.452
Not lobulated	118 (90.8%)	41 (85.4%)	
Lobulated	12 (9.2%)	7 (14.6%)	
Log_10_ (FSH) (IU/L)^g^	0.90 ± 0.43	1.07 ± 0.46	0.021^**^
FT3 (pmol/L)^h^	4.47 ± 0.88	4.16 ± 0.56	0.039^**^
WBC count (10^9^/L)^e^	5.77 ± 1.58	5.13 ± 1.17	0.007^**^
Monocyte percentage (%)^e^	7.18 ± 1.70	7.76 ± 1.56	0.019^**^
PT (s)^i^	11.30 ± 0.73	11.64 ± 0.80	0.002^**^
INR^i^	0.98 ± 0.06	1.01 ± 0.07	0.002^**^
Total bilirubin (μmol/L)^j^	13.12 ± 6.65	11.81 ± 4.65	0.307
Chlorine (mmol/L)^k^	102.29 ± 2.80	102.90 ± 3.62	0.078^*^

*PDH* Postoperative delayed hyponatremia, *PRL* Secreting, prolactin secreting, *GH* secreting Growth hormone secreting, *ACTH secreting* Adrenocorticotropic hormone secreting, *POD* Postoperative day, *FSH* Follicle-stimulating hormone, *FT3* Free triiodothyronine, *WBC* White blood cell, *PT* Prothrombin time, *INR* International normalized ratio

^*^Marginal statistical significance (*P* < 0.1)

^**^Statistical significance (*P* < 0.05)

^a^*n* = 5 missing

^b^*n* = 8 missing

^c^*n* = 15 missing

^d^*n* = 1 missing

^e^*n* = 7 missing

^f^*n* = 48 missing

^g^*n* = 23 missing

^h^*n* = 17 missing

^i^*n* = 18 missing

^j^*n* = 53 missing

^k^*n* = 18 missing

The origin datasets from training dataset were used to compare variables between patients with and without PDH. In the comparison, we filtered the variables with statistical significance (*P* < 0.05) or marginal significance (*P* < 0.1).

The proportions of hyponatremia on POD 1–2, visual impairment before operation, grade C-E of Hardy classification, and Hourglass sign were higher in patients with PDH compared to those without PDH. Furthermore, patients with PDH exhibited higher values of follicle stimulating hormone (FSH), monocyte percentage, PT, international normalized ratio (INR), and chlorine. Conversely, patients without PDH had higher free triiodothyronine (FT3) and white blood cell (WBC) count.

### Univariable and multivariable logistic regression analyses

In the univariable logistic regression analysis, we also selected the variables with statistical significance (*P* < 0.05) or marginal significance (*P* < 0.1). The results showed that hyponatremia on POD 1–2, visual impairment, FSH, FT3, WBC count, monocyte percentage, PT, INR, and total bilirubin were identified as influencing factors for PDH (Table 2).

**Table 2:** Univariable and multivariable logistic regression analyses based on the imputed datasets for the final model

Characteristics	Univariate analysis			Multivariate analysis		
	Coefficient	OR	*P*	Coefficient	OR	*P*
Age (year)	0.02	1.02	0.147			
Gender						
Female	Reference					
Male	− 0.14	0.87	0.646			
Clinical subtype						
Nonfunctioning	Reference					
PRL secreting	0.83	2.28	0.160			
GH secreting	− 0.05	0.95	0.898			
ACTH secreting	− 0.30	0.74	0.716			
PRL-GH secreting	− 0.22	0.80	0.852			
Preoperative hyponatremia						
No	Reference					
Yes	0.21	1.23	0.749			
Hyponatremia on POD 1–2						
No	Reference					
Yes	0.75	2.11	0.088^*^	0.97	2.64	0.033^**^
Polyuria						
No	Reference					
Yes	0.25	1.28	0.489			
Visual impairment						
No	Reference					
Yes	0.68	1.97	0.028^**^			
Hardy grade for suprasellar extension						
0	Reference					
A	− 0.71	0.49	0.160			
B	− 0.08	0.92	0.842			
C	0.57	1.76	0.175			
D	0.98	2.67	0.348			
E	1.39	4.01	0.150			
Hardy grade for sellar invasion						
Noninvasive	Reference					
Invasive	0.41	1.51	0.204			
Knosp grade						
Noninvasive	Reference					
Invasive	0.42	1.52	0.200			
Tumor shape 1						
In sella	Reference					
Ellipsoid	− 0.49	0.62	0.328			
Hourglass sign	0.25	1.29	0.517			
Tumor shape 2						
Not lobulated	Reference					
Lobulated	0.45	1.58	0.355			
Log_10_ (FSH) (IU/L)	0.84	2.32	0.020^**^			
FT3 (pmol/L)	− 0.54	0.58	0.012^**^			
WBC count (10^9^/L)	− 0.30	0.74	0.012^**^			
Monocyte percentage (%)	0.20	1.22	0.032^**^	0.20	1.22	0.047^**^
PT (s)	0.57	1.76	0.006^**^	0.58	1.78	0.008^**^
INR	5.42	226.26	0.017^**^			
Total bilirubin (μmol/L)	− 0.05	0.95	0.082^*^			
Chlorine (mmol/L)	0.06	1.06	0.277			

*PRL secreting* Prolactin secreting, *GH secreting* Growth hormone secreting, *ACTH secreting* Adrenocorticotropic hormone secreting, *POD* Postoperative day, *FSH* Follicle-stimulating hormone, *FT3* Free triiodothyronine, *WBC* White blood cell, *PT* Prothrombin time, *INR* International normalized ratio, *OR* Odds ratio

^*^Marginal statistical significance (*P* < 0.1)

^**^Statistical significance (*P* < 0.05)

The comparisons between with and without PDH groups, as well as the univariable logistic regression analysis, resulted in the filtering of 12 variables. Age and gender were also selected as covariates in the multivariable logistic regression analysis. Collinearity was detected between “visual impairment” and “Hardy grade for suprasellar extension” (Supplementary Table 5). As “Hardy grade for suprasellar extension” was one of the influencing factors for “visual impairment,” we removed “visual impairment” from the analysis. Collinearity was observed between “PT” and “INR”, and as “PT” is more frequently used in clinic, we removed the variable “INR” (Supplementary Table 5).

Based on the multivariable logistic regression analysis, hyponatremia on POD 1–2 (OR = 2.64, *P* = 0.033), the percentage of monocytes (OR = 1.22, *P* = 0.047), and PT (OR = 1.78, *P* = 0.008) were identified as risk factors for PDH (Table 2).

### Development and internal validation of the nomogram

**Fig. 1:**
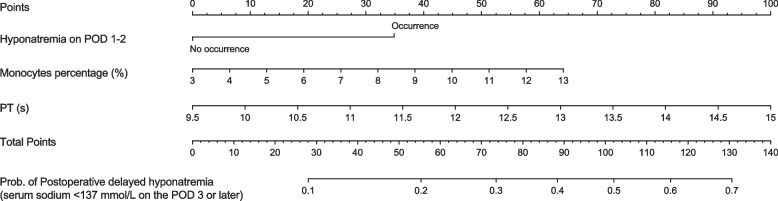
Nomogram for predicting the proportion of postoperative delayed hyponatremia (PDH) after endoscopic pituitary surgery in patients with pituitary adenomas. POD, post-operative day; T4, tetraiodothyronine; FSH, follicle stimulating hormone; PT, prothrombin time

Based on the predictors revealed in the multivariable logistic regression analysis, three variables were integrated into the final nomogram (complete dataset; Fig. 1). As there were 63 patients with PDH in the development set, the sample size met the requirement of the EVP = 10 criteria. The nomogram was available online (https://xiangmingcai.shinyapps.io/Delayed_Hyponatremia/). The ability of the nomogram model to discriminate patients likely to develop PDH was assessed with AUC values from the ROC curve analysis.

**Fig. 2:**
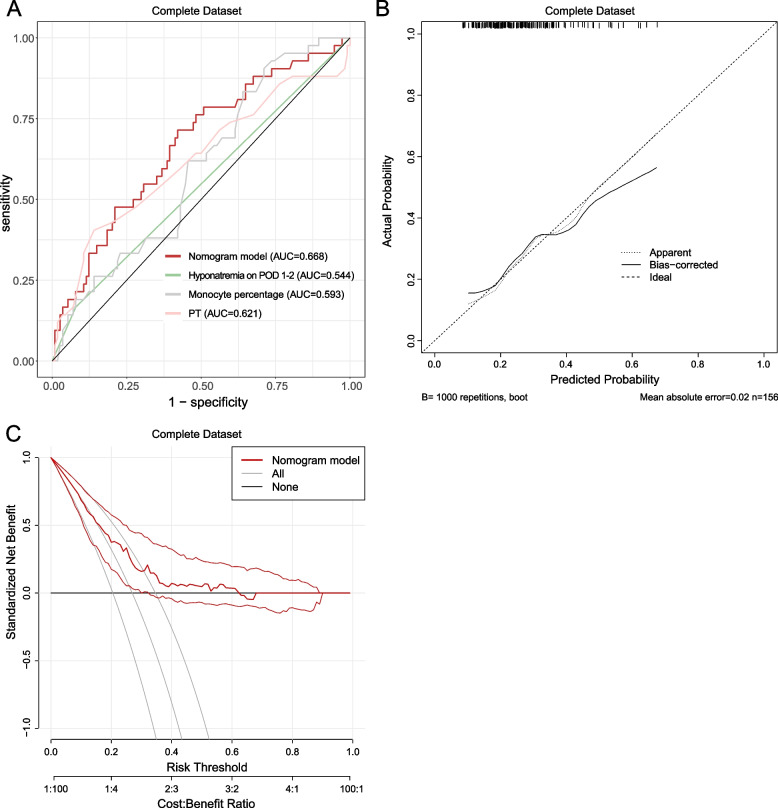
Predictive performance of the nomogram based on the complete dataset. **A** ROC analysis; **B** calibration plot; **C** decision curve analysis. AUC, area under the curve; PDH, postoperative delayed hyponatremia

The AUCs for the complete dataset and imputed datasets were as follows: 0.668 (0.571, 0.764), 0.650 (0.570, 0.731), 0.689 (0.613, 0.764), 0.668 (0.591, 0.746), 0.670 (0.591, 0.749), and 0.672 (0.595, 0.749) (Fig. 2A; Supplementary Fig. 3; Supplementary Table 6). Internal validation of the adjusted AUCs ranged from 0.613 to 0.688, which showed great discrimination (Supplementary Table 6). We further explored the performance of the nomogram model in various subgroups. Reliable and robust AUCs were observed in patients with different clinical subtypes (Supplementary Table 7). In the subgroup analysis of PDH severity, the nomogram demonstrated good discrimination ability for patients with mild to moderate PDH. However, poor predictive performance was detected in patients with severe PDH (Supplementary Table 7).

The calibration plots showed relatively adequate agreement of the predictive probability with actual observations (Fig. 2B; Supplementary Fig. 4). The Brier score showed a good overall model performance (0.182, 0.188, 0.183, 0.184, 0.184, and 0.186 for the complete dataset and imputed datasets 1–5, respectively). The DCA depicted the benefit derived from using the model in clinic. DCA results showed that if the threshold probability of PDH was between 0.2 and 0.6, utilizing the nomogram to screen patients with PDH provided a greater benefit compared to the assumption that all patients had PDH or that none of the patients had PDH (Fig. 2C; Supplementary Fig. 5). Also, because the incidence rate of PDH in the current research was 27.9%, between 0.2 and 0.6, application of the nomogram provides clinical benefit.

The results of external validation showed that for models derived from complete dataset and imputed datasets, the AUCs were 0.617, 0.610, 0.594, 0.612, 0.604, and 0.617 respectively. And these results suggested a good generalizability of the nomogram model.

## Discussion

PDH was a common cause of unexpected hospital readmissions after eTSS for PAs [3], and it can lead to headache, nausea, coma, and, in severe cases, even death [2]. Prediction of PDH will improve the patient-physician communication and further reduce the rates of hospital readmissions and complications. However, the predictors of PDH remain a topic of ongoing debate, and currently, there is no available clinical predictive online model for PDH prediction. In the current research, we explored the predictors of PDH and developed a reliable predictive dynamic online nomogram for predicting PDH.

The incidence rate of PDH varies widely among publications, ranging from 2 to 35% [1, 6]. One possible explanation for the wide range of incidence rates among different publications is the inconsistence in the diagnostic criteria used for PDH. In the current study, the incidence rate of PDH is 27.9%, which is relatively higher than that in some previous studies. We used a threshold of 137 mmol/L to define hyponatremia, as it is the normal range of serum sodium concentration applied in our institution. The threshold is slightly higher than 135 mmol/L used in some publications, which may lead to an increase incidence for PDH observed in our study.

Several predictors for PDH have been proposed. The predictive roles of age, gender, tumor type (functioning vs. nonfunctioning), and tumor size had been broadly discussed without any consistent conclusion [1]. Pooled results of a meta-analysis involving 13 studies showed that older age (over 55–60 years) was a statistically significant risk factor for PDH, whereas no significant associations were detected regarding gender, tumor type, or size [1]. In the current research, we did not find predictive value for age in both the univariable analysis (Table 1) and univariable regression analysis (Table 2). Some research has also reported potential predictors, such as sodium concentration on POD 1–3 [20–22], preoperative thyroid stimulating hormone (TSH) [5], body mass index [23], and postoperative diabetes insipidus [2, 6]. Due to the lack of examination, we did not evaluate diabetes insipidus. Instead, we analyzed the relationship between postoperative polyuria and PDH (Table 2). However, no statistical significance was detected for postoperative polyuria (OR = 1.28; *P* = 0.489).

Lin et al. explored predictive value of the shape of pituitary, including diaphragma sellae sinking depth, the deviation angle difference of the pituitary stalk, and postoperative length of the “measurable pituitary stalk” [6]. In the current study, we thoroughly analyzed shape-related factors, including tumor size, sellar barrier type, Hardy grade, Knosp grade, hourglass sign, and lobulated shape. Although a few of them showed statistical significance in univariate analysis, none of them was retained in the final multivariate regression model. Opposite opinions about the predictive roles of tumor size have been proposed [1]. Our results showed that among the lengths of maximum dimension, width, thickness, and height, none of them showed a significant difference between patients with and without PDH. Also, no predictive value of these variables was discovered in the univariable logistic regression analysis. Thus, based on our result, we concluded that tumor size was not a predictor for PDH. Overall, the predictors for PDH remain elusive. Further research is needed to verify our results with data from other medical centers.

The following hypotheses have been proposed to explain the occurrence of PDH: syndrome of inappropriate antidiuretic hormone secretion (SIADH), cerebral salt wasting syndrome (CSWS), glucocorticoid deficiency, hypothyroidism, and medications [1, 24]. However, no definitive consensus is currently available. Also, it remains unclear whether surgical injury, mass effect of tumor, or other factors can account for these hypotheses.

Previous studies have reported the predictive value of postoperative early sodium concentration for PDH. In a study conducted by Krogh et al., it was found that patients with PDH at POD 7 had significantly lower sodium levels during POD 1–2 [22]. Rajaratnam et al. revealed that patients with mean serum sodium levels > 138 mmol/L at POD 1–3 were unlikely to develop PDH (sensitivity, 55.2%; specificity, 83.9%) [20]. In the current study, hyponatremia on POD 1–2 showed a statistical significance in the multivariable analysis (OR = 2.64; *P* = 0.033; Table 2). Our results confirmed the opinion that hyponatremia on POD 1–2 is an independent risk factor for PDH. Further research should explore the potential mechanisms underlying the association between hyponatremia on POD 1–2 and PDH.

Hypothyroidism was found to be a possible reason of hyponatremia by decreasing water delivery to the nephron, leading to increased free water retention and inhibiting its excretion [5]. Tomita et al. found an interesting phenomenon that patients with higher yet normal preoperative TSH were more likely to develop PDH (*P* < 0.05) [5]. In our research, although no statistical significance was observed for preoperative TSH between patients with and without PDH, there is a trend indicating lower values of FT3 in patients with PDH (4.16 ± 0.56 vs. 4.47 ± 0.88, with vs. without PDH; *P* = 0.039; Table 1). However, in the multivariable logistic regression analysis, FT3 was excluded, suggesting that thyroid function may not play an important role in the development of PDH.

To our knowledge, the current research was the first investigation into the potential relationship between immune-related factors and PDH after eTSS for PAs. Our results showed that patients with a lower WBC count (5.13 ± 1.17 vs. 5.77 ± 1.58, with PDH vs. without PDH; *P* = 0.007; Table 1) and higher monocyte percentage (7.76 ± 1.56 vs. 7.18 ± 1.70, with PDH vs. without PDH; *P* = 0.019; Table 1) tend to develop PDH. The multivariable logistic regression analysis discovered statistical significance of monocyte percentage (OR = 1.22; *P* = 0.047; Table 2). In fact, there are little publication about immune factors and its relationship with the development of hyponatremia caused by impairment of pituitary. Although some case reports reported hyponatremia in immunotherapy-associated hypophysitis patients [25–27], it is difficult to find a corresponding role of immune factors in hypophysitis and pituitary adenoma. In the current research, the observed trend in WBC count and monocyte percentage may suggest a potentially less effective immune response to surgical injury, which in turn could have an impact on ADH secretion. However, this hypothesis needs to be further verified through well-designed clinical and experimental studies.

For the first time, our results showed that PT was an independent predictor of PDH (OR = 1.78; *P* = 0.008; Table 2). Also, the value of PT was significantly higher in patients with PDH compared to those without PDH (11.64 ± 0.80 vs. 11.30 ± 0.73, with PDH vs. without PDH; *P* = 0.002). The potential mechanism between PT and PDH could be attributed to the fact that higher PT levels reflect poor coagulation function. This, in return, may lead to more challenging surgery, prolonged operation time, and more severe impairment of the pituitary stalk. The impairment of the pituitary stalk can restrict the transport and secretion of ADH, potentially leading to PDH. In the research conducted by Lin et al., they investigated the predictive role of postoperative measurable pituitary stalk length for PDH. They suggested that change of pituitary stalk and damage to it may result in abnormal secretion of ADH and subsequently lead to PDH [6]. Consistent with their hypothesis, we also believe that the impairment of pituitary stalk acts as in intermediate factor between the predictor we identified and the development of PDH. Further studies are needed to investigate the relationship between PT and postoperative measurable pituitary stalk length and verify the predictive role of these factors in relation to PDH.

In the current study, we also conducted a subgroup analysis on the PDH severity to explore the suitable application scenarios for the nomogram. In clinic, neurosurgeons cannot differentiate between patients with different severities of PDH prior to its occurrence. Hence, we evaluated the predictive performance in “all PDH,” “moderate to severe PDH,” and “severe PDH” cases. Results showed a reliable performance in “all PDH” and “moderate to severe PDH” (AUCs, 0.609–0.706; Supplementary Table 7). However, the nomogram did not have any predictive ability for predicting severe PDH (Supplementary Table 7). Thus, the nomogram was only suitable for prediction of mild to moderate PDH.

Our study has several limitations. First, we were unable to include patients with thyrotropinoma and gonadotropinoma due to the absence of such cases in our center between January 2018 and October 2020. Also, although the nomogram was suitable for predicting mild to moderate PDH, it lacks the ability to predict the occurrence of severe PDH. Therefore, further research focused on prediction of severe PDH is necessary.

## Conclusion

Hyponatremia on POD 1–2, preoperative PT, and percentage of monocytes were identified as predictive factors of PDH. The predictive roles of PT and percentage of monocytes were identified for the first time. A dynamic nomogram is the first predictive model to identify patients with PDH after eTSS for PAs. These patients may benefit from early intervention and more monitoring.

## Supplementary Information

**Additional file 1: Supplementary Table 1.** TRIPOD checklist for the prediction model development and validation. **Supplementary Table 2.** Summary of missing data. **Supplementary Table 3.** Other characteristics of patients in the without PDH group and in the with PDH group. **Supplementary Table 4.** Univariable logistic regression analysis of the other characteristics. **Supplementary Table 5.** Spearman correlation analysis between some variables. **Supplementary Table 6.** Internal and external validation based on AUCs of the nomogram model in the complete dataset and 5 imputed datasets. **Supplementary Table 7.** Subgroup analysis based on AUCs of the nomogram model in the complete dataset and 5 imputed datasets. **Supplementary Figure 1.** The missing data patterns. Each row represents a missing pattern. Red and blue blocks indicate missing data and available data, respectively. The left y axis shows the number of missing data in the corresponding pattern. The right y axis shows the number of samples with the corresponding pattern. The bottom x axis shows the number of missing data for each variable. **Supplementary Figure 2.** The density plots of data before (black line) and after imputation (red line) show good imputation. **Supplementary Figure 3.** ROC analysis of the nomogram model and variables in the final model from the imputed dataset 1 (A), dataset 2 (B), dataset 3 (C), dataset 4 (D), and dataset 5 (E). AUC, area under the curve. **Supplementary Figure 4.** Calibration plots of the nomogram from the imputed dataset 1 (A), dataset 2 (B), dataset 3 (C), dataset4 (D), and dataset 5 (E). PDH, postoperative delayed hyponatremia. **Supplementary Figure 5.** Decision curve analysis with 95% confidence interval of the nomogram from the imputed dataset 1 (A), dataset 2 (B), dataset 3 (C), dataset 4 (D), and dataset 5 (E).

## Data Availability

The raw data of this article is available from the corresponding author upon reasonable request.
