# Supplementary material for: Predictors and dynamic online nomogram for postoperative delayed hyponatremia after endoscopic transsphenoidal surgery for pituitary adenomas: a single-center, retrospective, observational cohort study with external validation
Source: Chin Neurosurg J. 2023 Aug 1;9:19. doi: 10.1186/s41016-023-00334-3 (PMC10391999; doi:10.1186/s41016-023-00334-3)

Supplementary Table 1. TRIPOD checklist for prediction model development and validation

| **Section/Topic** | **Item** |  | **Checklist Item** | **Page** |
| --- | --- | --- | --- | --- |
| **Title and abstract** |  |  |  |  |
| Title | 1 | D;V | Identify the study as developing and/or validating a multivariable prediction model, the target population, and the outcome to be predicted. | 1 |
| Abstract | 2 | D;V | Provide a summary of objectives, study design, setting, participants, sample size, predictors, outcome, statistical analysis, results, and conclusions. | 2 |
| **Introduction** |  |  |  |  |
| Background and objectives | 3a | D;V | Explain the medical context (including whether diagnostic or prognostic) and rationale for developing or validating the multivariable prediction model, including references to existing models. | 3-4 |
|  | 3b | D;V | Specify the objectives, including whether the study describes the development or validation of the model or both. | 3-4 |
| **Methods** |  |  |  |  |
| Source of data | 4a | D;V | Describe the study design or source of data (e.g., randomized trial, cohort, or registry data), separately for the development and validation data sets, if applicable. | 4 |
|  | 4b | D;V | Specify the key study dates, including start of accrual; end of accrual; and, if applicable, end of follow-up. | 4 |
| Participants | 5a | D;V | Specify key elements of the study setting (e.g., primary care, secondary care, general population) including number and location of centres. | 4 |
|  | 5b | D;V | Describe eligibility criteria for participants. | 4 |
|  | 5c | D;V | Give details of treatments received, if relevant. | 4 |
| Outcome | 6a | D;V | Clearly define the outcome that is predicted by the prediction model, including how and when assessed. | 4-5 |
|  | 6b | D;V | Report any actions to blind assessment of the outcome to be predicted. | NA |
| Predictors | 7a | D;V | Clearly define all predictors used in developing or validating the multivariable prediction model, including how and when they were measured. | 4-5 |
|  | 7b | D;V | Report any actions to blind assessment of predictors for the outcome and other predictors. | NA |
| Sample size | 8 | D;V | Explain how the study size was arrived at. | 6 |
| Missing data | 9 | D;V | Describe how missing data were handled (e.g., complete-case analysis, single imputation, multiple imputation) with details of any imputation method. | 5-6 |
| Statistical analysis methods | 10a | D | Describe how predictors were handled in the analyses. | 5-7 |
|  | 10b | D | Specify type of model, all model-building procedures (including any predictor selection), and method for internal validation. | 5-7 |
|  | 10c | V | For validation, describe how the predictions were calculated. | 5-7 |
|  | 10d | D;V | Specify all measures used to assess model performance and, if relevant, to compare multiple models. | 5-7 |
|  | 10e | V | Describe any model updating (e.g., recalibration) arising from the validation, if done. | NA |
| Risk groups | 11 | D;V | Provide details on how risk groups were created, if done. | NA |
| Development vs. validation | 12 | V | For validation, identify any differences from the development data in setting, eligibility criteria, outcome, and predictors. | NA |
| **Results** |  |  |  |  |
| Participants | 13a | D;V | Describe the flow of participants through the study, including the number of participants with and without the outcome and, if applicable, a summary of the follow-up time. A diagram may be helpful. | NA |
|  | 13b | D;V | Describe the characteristics of the participants (basic demographics, clinical features, available predictors), including the number of participants with missing data for predictors and outcome. | 7-8 |
|  | 13c | V | For validation, show a comparison with the development data of the distribution of important variables (demographics, predictors and outcome). | NA |
| Model development | 14a | D | Specify the number of participants and outcome events in each analysis. | 5 |
|  | 14b | D | If done, report the unadjusted association between each candidate predictor and outcome. | 8-10 |
| Model specification | 15a | D | Present the full prediction model to allow predictions for individuals (i.e., all regression coefficients, and model intercept or baseline survival at a given time point). | Table 2 |
|  | 15b | D | Explain how to the use the prediction model. | Fig 1 |
| Model performance | 16 | D;V | Report performance measures (with CIs) for the prediction model. | 9-10/Fig 2, S3-5/Table 3, 4 |
| Model-updating | 17 | V | If done, report the results from any model updating (i.e., model specification, model performance). | NA |
| **Discussion** |  |  |  |  |
| Limitations | 18 | D;V | Discuss any limitations of the study (such as nonrepresentative sample, few events per predictor, missing data). | 14 |
| Interpretation | 19a | V | For validation, discuss the results with reference to performance in the development data, and any other validation data. | NA |
|  | 19b | D;V | Give an overall interpretation of the results, considering objectives, limitations, results from similar studies, and other relevant evidence. | 10-14 |
| Implications | 20 | D;V | Discuss the potential clinical use of the model and implications for future research. | 10-14 |
| **Other information** |  |  |  |  |
| Supplementary information | 21 | D;V | Provide information about the availability of supplementary resources, such as study protocol, Web calculator, and data sets. | 9-10 |
| Funding | 22 | D;V | Give the source of funding and the role of the funders for the present study. | 14 |

Items relevant only to the development of a prediction model are denoted by D, items relating solely to a validation of a prediction model are denoted by V, and items relating to both are denoted D;V. Some of the items were not applicable (NA) to the current study.

Supplementary Table 2. Summary of missing data.

| Variables | Missing Data | |
| --- | --- | --- |
|  | Count | Percentage |
| Age (year) | 0 | 0.00% |
| Gender | 0 | 0.00% |
| Female |  |  |
| Male |  |  |
| Clinical subtype | 5 | 2.21% |
| Nonfunctioning |  |  |
| PRL secreting |  |  |
| GH secreting |  |  |
| ACTH secreting |  |  |
| PRL-GH secreting |  |  |
| Preoperative hyponatremia | 8 | 3.54% |
| No |  |  |
| Yes |  |  |
| Hyponatremia on POD 1-2 | 15 | 6.64% |
| No |  |  |
| Yes |  |  |
| Polyuria | 0 | 0.00% |
| No |  |  |
| Yes |  |  |
| Visual impairment | 1 | 0.44% |
| No |  |  |
| Yes |  |  |
| Hardy grade for suprasellar extension | 8 | 3.54% |
| 0 |  |  |
| A |  |  |
| B |  |  |
| C |  |  |
| D |  |  |
| E |  |  |
| Hardy grade for sellar invasion | 8 | 3.54% |
| Noninvasive |  |  |
| Invasive |  |  |
| Knosp grade | 7 | 3.10% |
| Noninvasive |  |  |
| Invasive |  |  |
| Tumor shape 1 | 48 | 21.24% |
| In sella |  |  |
| Ellipsoid |  |  |
| Hourglass sign |  |  |
| Tumor shape 2 | 48 | 21.24% |
| Not lobulated |  |  |
| Lobulated |  |  |
| Log_10_ (FSH) (IU/L) | 23 | 10.18% |
| FT3 (pmol/L) | 17 | 7.52% |
| WBC count (10^9^/L) | 7 | 3.10% |
| Monocyte percentage (%) | 7 | 3.10% |
| PT (s) | 18 | 7.96% |
| INR | 18 | 7.96% |
| Chlorine (mmol/L) | 9 | 3.98% |
|  |  |  |
| Primary-recurrence subtype | 0 | 0.00% |
| Primary |  |  |
| Recurrence |  |  |
| History of pituitary surgery | 0 | 0.00% |
| No |  |  |
| Yes |  |  |
| History of medication | 0 | 0.00% |
| No |  |  |
| Yes |  |  |
| History of radiotherapy | 0 | 0.00% |
| No |  |  |
| Yes |  |  |
| Headache | 0 | 0.00% |
| No |  |  |
| Yes |  |  |
| Visual field defect | 0 | 0.00% |
| No |  |  |
| Yes |  |  |
| Moon face | 0 | 0.00% |
| No |  |  |
| Yes |  |  |
| Acromegalia | 0 | 0.00% |
| No |  |  |
| Yes |  |  |
| Lengths of tumor maximum dimension (mm) | 24 | 10.62% |
| Lengths of tumor height (mm) | 48 | 21.24% |
| Lengths of tumor width (mm) | 48 | 21.24% |
| Lengths of tumor thickness (mm) | 49 | 21.68% |
| Sellar barrier | 48 | 21.24% |
| Weak |  |  |
| Strong |  |  |
| Tumor signal intensity | 50 | 22.12% |
| Lower |  |  |
| Equal |  |  |
| Higher |  |  |
| Optic nerve compression | 8 | 3.54% |
| No |  |  |
| Yes |  |  |
| Pituitary apoplexy | 7 | 3.10% |
| No |  |  |
| Yes |  |  |
| Residual tumor | 51 | 22.57% |
| No |  |  |
| Yes |  |  |
| Log_10_ (Prolacin) (mIU/L) | 15 | 6.64% |
| Testosterone | 41 | 18.14% |
| <=3 nmol/L |  |  |
| >3 nmol/L |  |  |
| Log_10_ (Estradiol) (pmol/L) | 29 | 12.83% |
| Log_10_ (Progesterone) (nmol/L) | 44 | 19.47% |
| Log_10_ (LH) (IU/L) | 22 | 9.73% |
| Log_10_ (TSH) (mIU/L) | 17 | 7.52% |
| T3 (nmol/L) | 18 | 7.96% |
| T4 (nmol/L) | 17 | 7.52% |
| FT4 (pmol/L) | 17 | 7.52% |
| Log_10_ (ACTH) (pg/ml) | 21 | 9.29% |
| Cortisol (μmol/L) | 15 | 6.64% |
| Log_10_ (IGF-1) (ng/ml) | 16 | 7.08% |
| Log_10_ (GH) (μg/L) | 14 | 6.19% |
| Neutrophil percentage (%) | 7 | 3.10% |
| Lymphocyte percentage (%) | 7 | 3.10% |
| Basophil percentage | 7 | 3.10% |
| <=1 % |  |  |
| >1 % |  |  |
| Log_10_ (Eosinophil percentage) (%) | 7 | 3.10% |
| RBC count (10^12^/L) | 7 | 3.10% |
| HCT (%) | 7 | 3.10% |
| Log_10_ (RDW) (%) | 7 | 3.10% |
| Hemoglobin (g/L) | 7 | 3.10% |
| MCV (fL) | 7 | 3.10% |
| MCH (pg) | 7 | 3.10% |
| MCHC (g/L) | 7 | 3.10% |
| Platelet count (10^9^/L) | 7 | 3.10% |
| MPV (fL) | 7 | 3.10% |
| Thrombocytocrit (%) | 7 | 3.10% |
| TT (s) | 18 | 7.96% |
| APTT (s) | 18 | 7.96% |
| Log_10_ (Fibrinogen) (g/L) | 18 | 7.96% |
| Total protein (g/L) | 29 | 12.83% |
| Albumin (g/L) | 29 | 12.83% |
| Globulin (g/L) | 29 | 12.83% |
| Log_10_ (ALT) (U/L) | 8 | 3.54% |
| Log_10_ (AST) (U/L) | 56 | 24.78% |
| Total bilirubin (μmol/L) | 53 | 23.45% |
| Potassium (mmol/L) | 8 | 3.54% |
| Creatinine (μmol/L) | 9 | 3.98% |
| Urea (mmol/L) | 9 | 3.98% |
| Uric acid (μmol/L) | 32 | 14.16% |
| CRP | 46 | 20.35% |
| <=8 mg/L |  |  |
| >8 mg/L |  |  |

PRL secreting, prolactin secreting; GH secreting, growth hormone secreting; ACTH secreting, adrenocorticotropic hormone secreting; POD, post-operative day; FSH, follicle stimulating hormone; FT3, free triiodothyronine; WBC, white blood cell; PT, prothrombin time; INR, international normalized ratio; LH, luteinizing hormone; TSH, thyroid-stimulating hormone; T3, triiodothyronine; T4, tetraiodothyronine; FT4, free tetraiodothyronine; IGF-1, insulin-like growth factor-1; RBC, red blood cell; HCT, hematocrit; RDW, red blood cell distribution width; MCV, mean corpuscular volume; MCH, mean corpuscular hemoglobin; MCHC, mean corpuscular hemoglobin concentration; MPV, mean platelet volume; TT, thrombin time; APTT, activated partial thromboplastin time; ALT, alanine aminotransferase; AST, aspartate transaminase; CRP, C-reactive protein;

Supplementary Table 3. Other characteristics of patients in the without PDH group and in the with PDH group.

| Variables | Without PDH | With PDH | *P* |
| --- | --- | --- | --- |
|  | (N = 163) | (N = 63) |  |
| Primary-recurrence subtype |  |  | 0.304 |
| Primary | 134 (82.2%) | 56 (88.9%) |  |
| Recurrence | 29 (17.8%) | 7 (11.1%) |  |
| History of pituitary surgery |  |  | 0.406 |
| No | 133 (81.6%) | 55 (87.3%) |  |
| Yes | 30 (18.4%) | 8 (12.7%) |  |
| History of medication |  |  | 1.000 |
| No | 156 (95.7%) | 60 (95.2%) |  |
| Yes | 7 (4.3%) | 3 (4.8%) |  |
| History of radiotherapy |  |  | 0.279 |
| No | 163 (100.0%) | 62 (98.4%) |  |
| Yes | 0 (0.0%) | 1 (1.6%) |  |
| Headache |  |  | 0.813 |
| No | 108 (66.3%) | 40 (63.5%) |  |
| Yes | 55 (33.7%) | 23 (36.5%) |  |
| Visual field defect |  |  | 0.312 |
| No | 119 (73.0%) | 41 (65.1%) |  |
| Yes | 44 (27.0%) | 22 (34.9%) |  |
| Moon face |  |  | 1.000 |
| No | 157 (96.3%) | 61 (96.8%) |  |
| Yes | 6 (3.7%) | 2 (3.2%) |  |
| Acromegalia |  |  | 1.000 |
| No | 134 (82.2%) | 52 (82.5%) |  |
| Yes | 29 (17.8%) | 11 (17.5%) |  |
| Lengths of tumor maximum dimension (mm) ^a^ | 25.71 ± 8.92 | 27.46 ± 12.16 | 0.339 |
| Lengths of tumor height (mm) ^b^ | 21.79 ± 9.24 | 24.87 ± 11.84 | 0.117 |
| Lengths of tumor width (mm) ^b^ | 22.81 ± 7.04 | 23.46 ± 8.66 | 0.642 |
| Lengths of tumor thickness (mm) ^c^ | 19.22 ± 6.86 | 20.41 ± 8.98 | 0.596 |
| Sellar barrier ^b^ |  |  | 0.570 |
| Weak | 54 (41.5%) | 17 (35.4%) |  |
| Strong | 76 (58.5%) | 31 (64.6%) |  |
| Tumor signal intensity ^d^ |  |  | 0.526 |
| Lower | 20 (15.6%) | 6 (12.5%) |  |
| Equal | 76 (59.4%) | 26 (54.2%) |  |
| Higher | 32 (25.0%) | 16 (33.3%) |  |
| Optic nerve compression ^e^ |  |  | 0.741 |
| No | 57 (36.3%) | 20 (32.8%) |  |
| Yes | 100 (63.7%) | 41 (67.2%) |  |
| Pituitary apoplexy ^f^ |  |  | 0.823 |
| No | 115 (72.8%) | 46 (75.4%) |  |
| Yes | 43 (27.2%) | 15 (24.6%) |  |
| Residual tumor ^g^ |  |  | 0.273 |
| No | 100 (83.3%) | 50 (90.9%) |  |
| Yes | 20 (16.7%) | 5 (9.1%) |  |
| Log_10_ (Prolacin) (mIU/L) ^h^ | 2.55 ± 0.42 | 2.60 ± 0.43 | 0.767 |
| Testosterone ^i^ |  |  | 0.855 |
| <=3 nmol/L | 74 (56.5%) | 32 (59.3%) |  |
| >3 nmol/L | 57 (43.5%) | 22 (40.7%) |  |
| Log_10_ (Estradiol) (pmol/L) ^j^ | 2.05 ± 0.44 | 2.03 ± 0.39 | 0.918 |
| Log_10_ (Progesterone) (nmol/L) ^k^ | 0.08 ± 0.58 | 0.04 ± 0.63 | 0.863 |
| Log_10_ (LH) (IU/L) ^l^ | 0.47 ± 0.52 | 0.54 ± 0.61 | 0.209 |
| Log_10_ (TSH) (mIU/L) ^m^ | 0.17 ± 0.40 | 0.26 ± 0.32 | 0.263 |
| T3 (nmol/L) ^n^ | 1.31 ± 0.37 | 1.24 ± 0.32 | 0.122 |
| T4 (nmol/L) ^m^ | 101.55 ± 25.42 | 95.43 ± 23.33 | 0.162 |
| FT4 (pmol/L) ^m^ | 9.92 ± 2.86 | 9.44 ± 2.32 | 0.360 |
| Log_10_ (ACTH) (pg/ml) ^o^ | 1.40 ± 0.26 | 1.41 ± 0.25 | 0.617 |
| Cortisol (μmol/L) ^h^ | 0.31 ± 0.15 | 0.30 ± 0.14 | 0.970 |
| Log_10_ (GH) (μg/L) ^p^ | -0.52 ± 0.99 | -0.62 ± 0.89 | 0.668 |
| Log_10_ (IGF-1) (ng/ml) ^q^ | 2.23 ± 0.37 | 2.16 ± 0.37 | 0.143 |
| Neutrophil percentage (%) ^f^ | 54.58 ± 9.56 | 53.64 ± 9.16 | 0.504 |
| Lymphocyte percentage (%) ^f^ | 35.38 ± 8.89 | 35.47 ± 8.66 | 0.944 |
| Basophil percentage ^f^ |  |  | 0.223 |
| <=1 % | 154 (97.5%) | 57 (93.4%) |  |
| >1 % | 4 (2.5%) | 4 (6.6%) |  |
| Log_10_ (Eosinophil percentage) (%) ^f^ | 0.27 ± 0.46 | 0.35 ± 0.27 | 0.271 |
| RBC count (10^12^/L) ^f^ | 4.43 ± 0.49 | 4.34 ± 0.54 | 0.260 |
| HCT (%) ^f^ | 0.40 ± 0.04 | 0.39 ± 0.04 | 0.195 |
| Log_10_ (RDW) (%) ^f^ | 1.11 ± 0.04 | 1.12 ± 0.03 | 0.503 |
| Hemoglobin (g/L) ^f^ | 133.01 ± 15.19 | 129.93 ± 15.48 | 0.188 |
| MCV (fL) ^f^ | 89.86 ± 5.45 | 89.73 ± 4.65 | 0.432 |
| MCH (pg) ^f^ | 30.09 ± 2.15 | 30.01 ± 1.69 | 0.240 |
| MCHC (g/L) ^f^ | 334.83 ± 12.93 | 334.56 ± 12.13 | 0.560 |
| Platelet count (10^9^/L) ^f^ | 206.78 ± 49.30 | 201.28 ± 67.31 | 0.243 |
| MPV (fL) ^f^ | 10.80 ± 1.20 | 10.86 ± 1.44 | 0.877 |
| Thrombocytocrit (%) ^f^ | 0.22 ± 0.05 | 0.21 ± 0.06 | 0.107 |
| TT (s) ^n^ | 17.18 ± 1.30 | 17.35 ± 1.22 | 0.432 |
| APTT (s) ^n^ | 26.70 ± 3.66 | 26.89 ± 3.35 | 0.717 |
| Log_10_ (Fibrinogen) (g/L) ^n^ | 0.42 ± 0.09 | 0.41 ± 0.10 | 0.349 |
| Total protein (g/L) ^j^ | 69.02 ± 5.89 | 68.87 ± 6.34 | 0.708 |
| Albumin (g/L) ^j^ | 39.94 ± 4.14 | 39.45 ± 3.86 | 0.439 |
| Globulin (g/L) ^j^ | 29.08 ± 3.41 | 29.42 ± 4.12 | 0.996 |
| Log_10_ (ALT) (U/L) ^e^ | 1.49 ± 0.22 | 1.45 ± 0.18 | 0.218 |
| Log_10_ (AST) (U/L) ^r^ | 1.38 ± 0.18 | 1.36 ± 0.14 | 0.804 |
| Potassium (mmol/L) ^e^ | 4.12 ± 0.36 | 4.05 ± 0.34 | 0.186 |
| Creatinine (μmol/L) ^s^ | 60.22 ± 15.74 | 61.26 ± 13.98 | 0.632 |
| Urea (mmol/L) ^s^ | 5.14 ± 1.30 | 5.48 ± 1.61 | 0.245 |
| Uric acid (μmol/L) ^u^ | 315.54 ± 88.97 | 304.87 ± 78.78 | 0.559 |
| CRP ^v^ |  |  | 0.414 |
| <=8 mg/L | 124 (96.9%) | 49 (94.2%) |  |
| >8 mg/L | 4 (3.1%) | 3 (5.8%) |  |

PDH, postoperative delayed hyponatremia; LH, luteinizing hormone; TSH, thyroid-stimulating hormone; T3, triiodothyronine; T4, tetraiodothyronine; FT4, free tetraiodothyronine; ACTH, adrenocorticotropic hormone; GH, growth hormone; IGF-1, insulin-like growth factor-1; RBC, red blood cell; HCT, haematocrit; RDW, red blood cell distribution width; MCV, mean corpuscular volume; MCH, mean corpuscular hemoglobin; MCHC, mean corpuscular hemoglobin concentration; MPV, mean platelet volume; TT, thrombin time; APTT, activated partial thromboplastin time; ALT, alanine aminotransferase; AST, aspartate transaminase; CRP, C-reactive protein; ^a^ n = 24 missing. ^b^ n = 48 missing. ^c^ n = 49 missing. ^d^ n = 50 missing. ^e^ n = 8 missing. ^f^ n = 7 missing. ^g^ n = 51 missing. ^h^ n = 15 missing. ^i^ n = 41 missing. ^j^ n = 29 missing. ^k^ n = 44 missing. ^l^ n = 22 missing. ^m^ n = 17 missing. ^n^ n = 18 missing. ^o^ n = 21 missing. ^p^ n = 14 missing. ^q^ n = 16 missing. ^r^ n = 56 missing. ^s^ n = 9 missing. ^u^ n = 32 missing. ^v^ n = 46 missing.

Supplementary Table 4. Univariate logistic regression analysis of other characteristics.

| Variables | Coefficient | OR | *P* |
| --- | --- | --- | --- |
| Primary-recurrence subtype |  |  |  |
| Primary | Reference |  |  |
| Recurrence | -0.55 | 0.58 | 0.224 |
| History of pituitary surgery |  |  |  |
| No | Reference |  |  |
| Yes | -0.44 | 0.65 | 0.308 |
| History of medication |  |  |  |
| No | Reference |  |  |
| Yes | 0.11 | 1.11 | 0.878 |
| History of radiotherapy |  |  |  |
| No | Reference |  |  |
| Yes | 15.53 | 5.57×10^6^ | 0.986 |
| Headache |  |  |  |
| No | Reference |  |  |
| Yes | 0.12 | 1.13 | 0.695 |
| Visual field defect |  |  |  |
| No | Reference |  |  |
| Yes | 0.37 | 1.45 | 0.242 |
| Moon face |  |  |  |
| No | Reference |  |  |
| Yes | -0.15 | 0.86 | 0.854 |
| Acromegalia |  |  |  |
| No | Reference |  |  |
| Yes | -0.02 | 0.98 | 0.953 |
| Lengths of tumor maximum dimension (mm) | 0.01 | 1.01 | 0.418 |
| Lengths of tumor height (mm) | 0.02 | 1.02 | 0.136 |
| Lengths of tumor width (mm) | <0.01 | 1.00 | 0.926 |
| Lengths of tumor thickness (mm) | 0.01 | 1.01 | 0.601 |
| Sellar barrier |  |  |  |
| Weak | Reference |  |  |
| Strong | 0.12 | 1.13 | 0.712 |
| Tumor signal intensity |  |  |  |
| Lower | Reference |  |  |
| Equal | 0.03 | 1.03 | 0.961 |
| Higher | 0.44 | 1.56 | 0.393 |
| Optic nerve compression |  |  |  |
| No | Reference |  |  |
| Yes | 0.08 | 1.08 | 0.804 |
| Pituitary apoplexy |  |  |  |
| No | Reference |  |  |
| Yes | -0.16 | 0.86 | 0.654 |
| Residual tumor |  |  |  |
| No | Reference |  |  |
| Yes | -0.74 | 0.48 | 0.132 |
| Log_10_ (Prolacin) (mIU/L) | 0.18 | 1.19 | 0.632 |
| Testosterone |  |  |  |
| <=3 nmol/L | Reference |  |  |
| >3 nmol/L | -0.07 | 0.93 | 0.821 |
| Log_10_ (Estradiol) (pmol/L) | -0.28 | 0.75 | 0.478 |
| Log_10_ (Progesterone) (nmol/L) | -0.12 | 0.89 | 0.647 |
| Log_10_ (LH) (IU/L) | 0.18 | 1.20 | 0.519 |
| Log_10_ (TSH) (mIU/L) | 0.60 | 1.83 | 0.166 |
| T3 (nmol/L) | -0.49 | 0.61 | 0.285 |
| T4 (nmol/L) | -0.01 | 0.99 | 0.127 |
| FT4 (pmol/L) | -0.06 | 0.94 | 0.300 |
| Log_10_ (ACTH) (pg/ml) | 0.12 | 1.13 | 0.840 |
| Cortisol (μmol/L) | -0.30 | 0.74 | 0.776 |
| Log_10_ (GH) (μg/L) | -0.11 | 0.90 | 0.486 |
| Log_10_ (IGF-1) (ng/ml) | -0.49 | 0.62 | 0.231 |
| Neutrophil percentage (%) | -0.01 | 0.99 | 0.480 |
| Lymphocyte percentage (%) | <0.01 | 1.00 | 0.825 |
| Basophil percentage |  |  |  |
| <=1 % | Reference |  |  |
| >1 % | 0.68 | 1.98 | 0.329 |
| Log_10_ (Eosinophil percentage) (%) | 0.37 | 1.45 | 0.482 |
| RBC count (10^12^/L) | -0.33 | 0.72 | 0.260 |
| HCT (%) | -5.38 | 0.01 | 0.146 |
| Log_10_ (RDW) (%) | 1.30 | 3.67 | 0.732 |
| Hemoglobin (g/L) | -0.01 | 0.99 | 0.156 |
| MCV (fL) | -0.01 | 0.99 | 0.779 |
| MCH (pg) | -0.02 | 0.98 | 0.767 |
| MCHC (g/L) | <0.01 | 1.00 | 0.971 |
| Platelet count (10^9^/L) | >-0.01 | 1.00 | 0.469 |
| MPV (fL) | 0.03 | 1.04 | 0.779 |
| Thrombocytocrit (%) | -2.80 | 0.06 | 0.333 |
| TT (s) | 0.08 | 1.09 | 0.458 |
| APTT (s) | 0.02 | 1.02 | 0.672 |
| Log_10_ (Fibrinogen) (g/L) | -0.75 | 0.47 | 0.635 |
| Total protein (g/L) | -0.01 | 1.00 | 0.866 |
| Albumin (g/L) | -0.02 | 0.98 | 0.575 |
| Globulin (g/L) | 0.03 | 1.03 | 0.498 |
| Log_10_ (ALT) (U/L) | -0.75 | 0.47 | 0.309 |
| Log_10_ (AST) (U/L) | -0.84 | 0.43 | 0.399 |
| Potassium (mmol/L) | -0.57 | 0.56 | 0.193 |
| Creatinine (μmol/L) | 0.00 | 1.00 | 0.815 |
| Urea (mmol/L) | 0.16 | 1.17 | 0.154 |
| Uric acid (μmol/L) | >-0.01 | 1.00 | 0.474 |
| CRP |  |  |  |
| <=8 mg/L | Reference |  |  |
| >8 mg/L | 0.82 | 2.26 | 0.283 |

LH, luteinizing hormone; TSH, thyroid-stimulating hormone; T3, triiodothyronine; T4, tetraiodothyronine; FT4, free tetraiodothyronine; ACTH, adrenocorticotropic hormone; GH, growth hormone; IGF-1, insulin-like growth factor-1; RBC, red blood cell; HCT, hematocrit; RDW, red blood cell distribution width; MCV, mean corpuscular volume; MCH, mean corpuscular hemoglobin; MCHC, mean corpuscular hemoglobin concentration; MPV, mean platelet volume; TT, thrombin time; APTT, activated partial thromboplastin time; ALT, alanine aminotransferase; AST, aspartate transaminase; CRP, C-reactive protein.

Supplementary Table 5. Spearman correlation analysis between some variables.

| Variable 1 | Variable 2 | R value between variable 1 and variable 2 |
| --- | --- | --- |
| Visual impairment | Hardy grade for suprasellar extension | 0.363** |
| PT | INR | 1.000** |

INR, international normalized ratio; PT, prothrombin time. **Statistical significance (*P* <0.01).

Supplementary Table 6. Internal validation based on AUCs of the nomogram model in complete dataset and 5 imputed datasets.

| AUC | Unadjusted | 3-fold cross validation | 5-fold cross validation | 10-fold cross validation | Jackknife validation | Bootstrap validation |
| --- | --- | --- | --- | --- | --- | --- |
| Complete Dataset | 0.668 | 0.627 | 0.637 | 0.666 | 0.613 | 0.665 |
| Imputed Dataset 1 | 0.650 | 0.629 | 0.628 | 0.649 | 0.613 | 0.649 |
| Imputed Dataset 2 | 0.689 | 0.672 | 0.672 | 0.681 | 0.659 | 0.688 |
| Imputed Dataset 3 | 0.668 | 0.649 | 0.649 | 0.660 | 0.636 | 0.667 |
| Imputed Dataset 4 | 0.670 | 0.652 | 0.653 | 0.665 | 0.640 | 0.669 |
| Imputed Dataset 5 | 0.672 | 0.654 | 0.654 | 0.658 | 0.640 | 0.670 |

AUC, area under curve.

Supplementary Table 7. Subgroup analysis based on AUCs of the nomogram model in complete dataset and 5 imputed datasets.

| Subgroup | Complete dataset | |  | Imputed Dataset 1 | |  | Imputed Dataset 2 | |  | Imputed Dataset 3 | |  | Imputed Dataset 4 | |  | Imputed Dataset 5 | |
| --- | --- | --- | --- | --- | --- | --- | --- | --- | --- | --- | --- | --- | --- | --- | --- | --- | --- |
|  | AUC | Count |  | AUC | Count |  | AUC | Count |  | AUC | Count |  | AUC | Count |  | AUC | Count |
| PDH severity subgroups |  |  |  |  |  |  |  |  |  |  |  |  |  |  |  |  |  |
| All PDH | 0.668** | 42/114 |  | 0.650** | 63/163 |  | 0.689** | 63/163 |  | 0.668** | 63/163 |  | 0.670** | 63/163 |  | 0.672** | 63/163 |
| Moderate to severe PDH | 0.582 | 10/146 |  | 0.593 | 18/208 |  | 0.657* | 18/208 |  | 0.643* | 18/208 |  | 0.605 | 18/208 |  | 0.614 | 18/208 |
| Severe PDH | 0.531 | 5/151 |  | 0.503 | 10/216 |  | 0.601 | 10/216 |  | 0.596 | 10/216 |  | 0.524 | 10/216 |  | 0.531 | 10/216 |
| Clinical subtypes |  |  |  |  |  |  |  |  |  |  |  |  |  |  |  |  |  |
| Nonfunctioning PAs | 0.574 | 27/80 |  | 0.581 | 42/110 |  | 0.600 | 42/110 |  | 0.597 | 42/111 |  | 0.599 | 42/110 |  | 0.579 | 42/108 |
| PRL secreting PAs | 1.000 | 4/2 |  | 0.976** | 6/7 |  | 0.976** | 6/7 |  | 0.976** | 6/7 |  | 0.979** | 6/8 |  | 0.976** | 6/7 |
| GH secreting PAs | 0.807* | 8/22 |  | 0.691* | 12/36 |  | 0.819** | 12/36 |  | 0.720* | 12/35 |  | 0.717** | 12/35 |  | 0.762** | 12/36 |
| ACTH secreting PAs | 0.833 | 2/6 |  | 0.857 | 2/7 |  | 0.857 | 2/7 |  | 0.857 | 2/7 |  | 0.857 | 2/7 |  | 0.889 | 2/9 |
| PRL-GH secreting PAs | 1.000 | 1/3 |  | 1.000 | 1/3 |  | 1.000 | 1/3 |  | 1.000 | 1/3 |  | 1.000 | 1/3 |  | 1.000 | 1/3 |

AUC, area under curve. PDH, postoperative delayed hyponatremia; PRL secreting, prolactin secreting; GH secreting, growth hormone secreting; ACTH secreting, adrenocorticotropic hormone secreting; PAs, pituitary adenomas. **P* ≤ 0.05; ***P* ≤ 0.01. Count: number of samples with/without PDH. Moderate to severe PDH: serum sodium concentration <130 mmol/L. Severe PDH: serum sodium concentration <125 mmol/L.

**Supplementary Figure 1**. The missing data patterns.


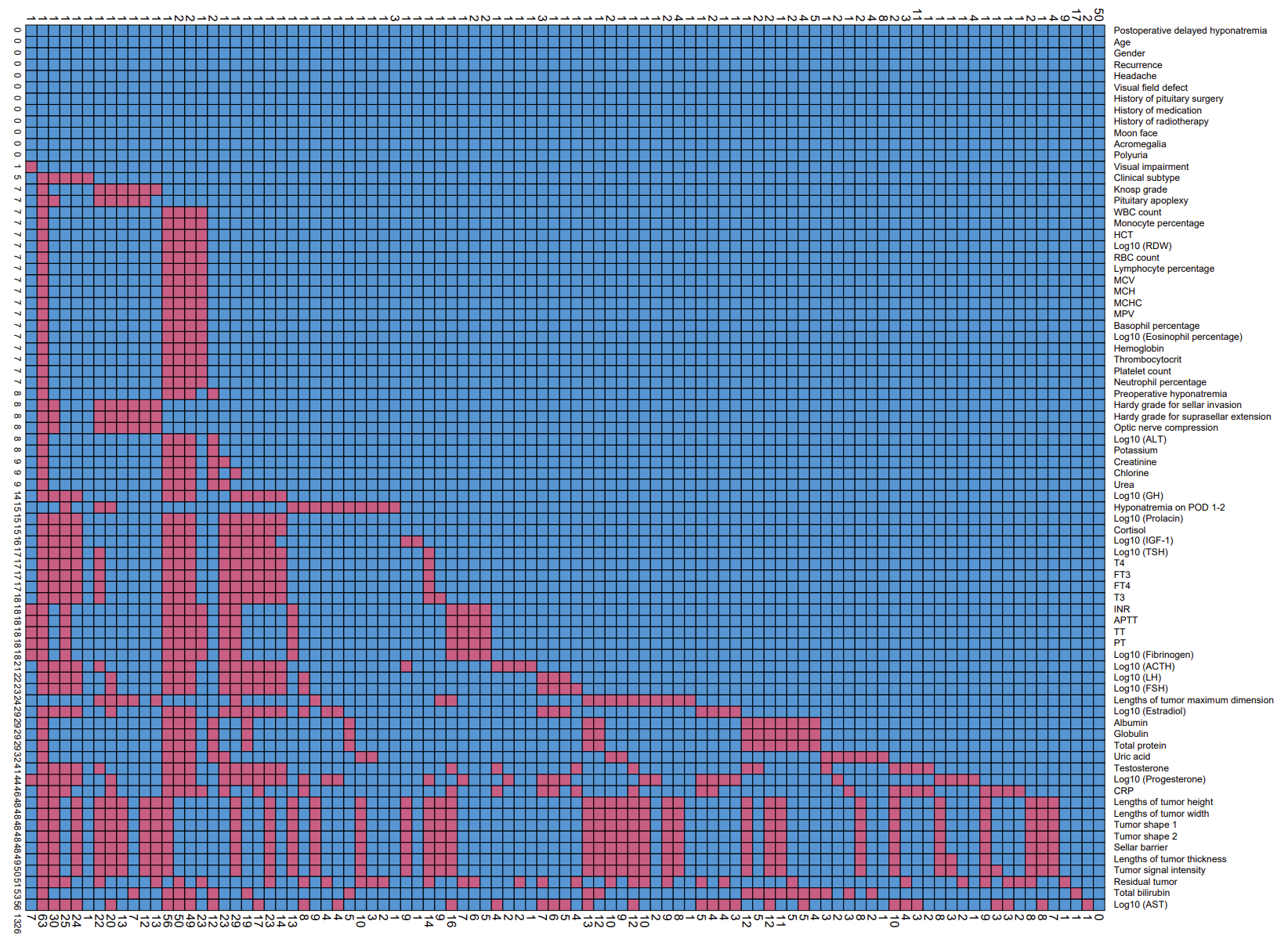


**Supplementary Figure 2**. The density plots of data before (black line) and after imputation (red line) show good imputation.


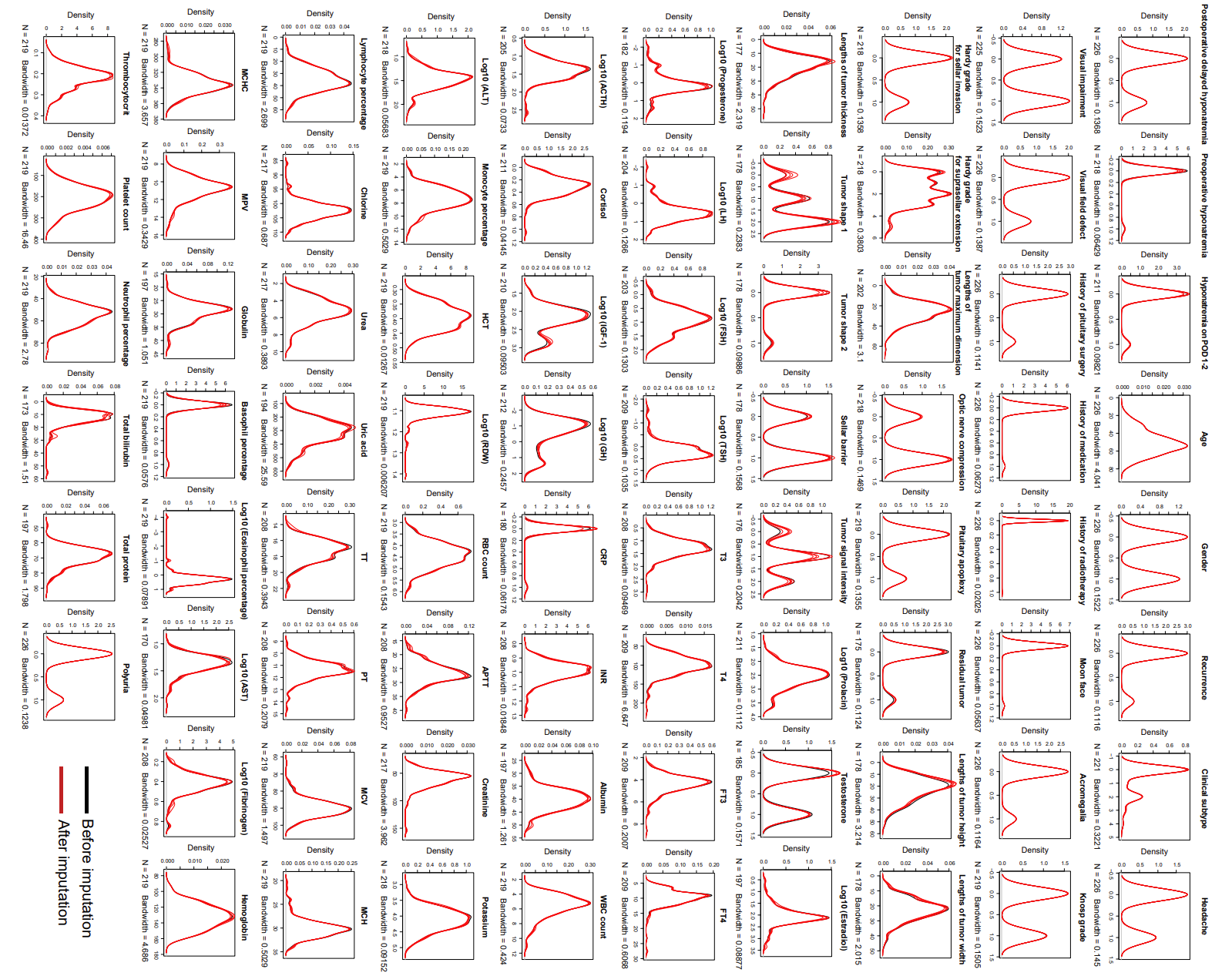


**Supplementary Figure 3**. ROC analysis of the nomogram model and variables in the final model from the imputed dataset 1 (**A**), dataset 2 (**B**), dataset 3 (**C**), dataset 4 (**D**), and dataset 5 (**E**). AUC, area under the curve.


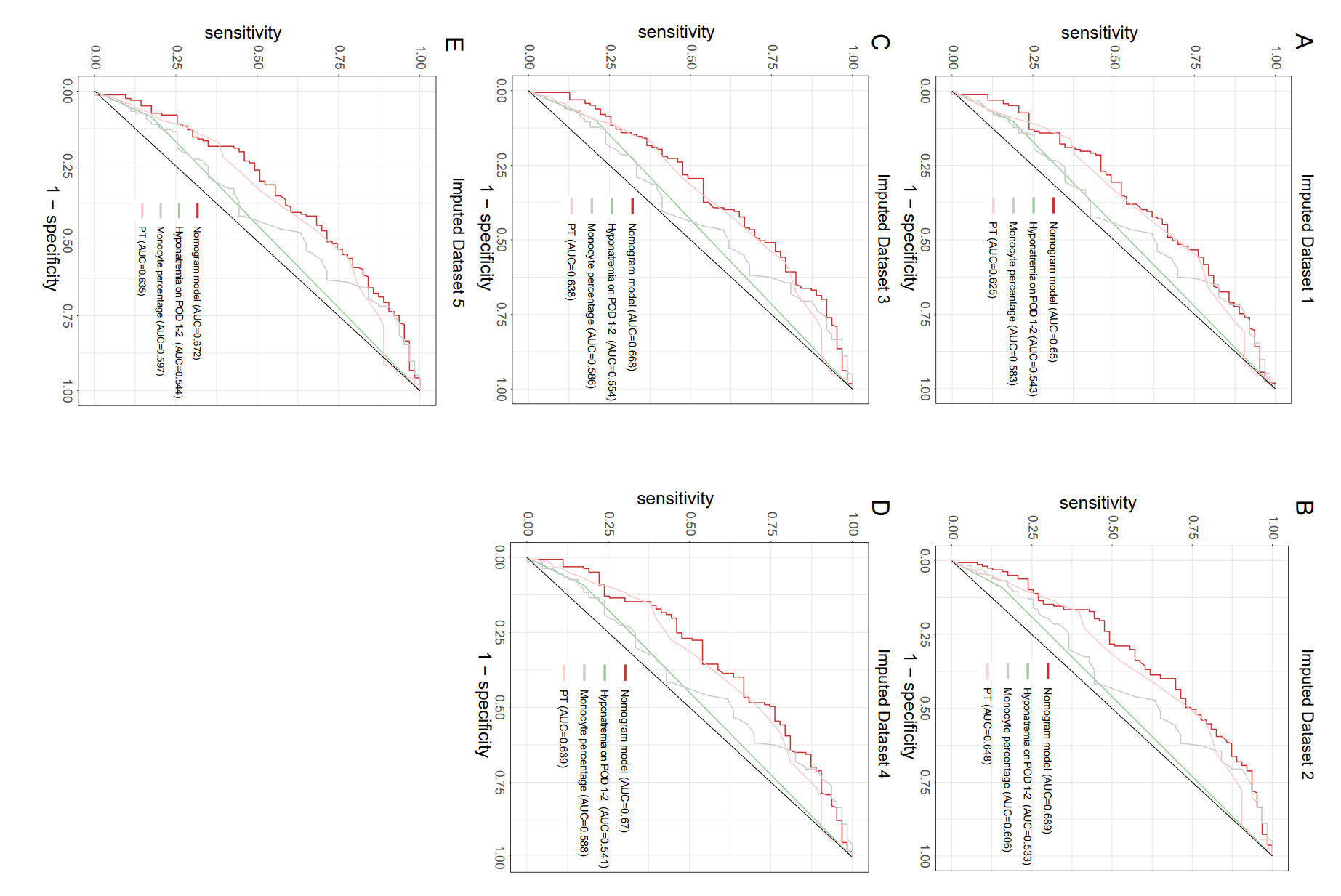


**Supplementary Figure 4**. Calibration plots of the nomogram from the imputed dataset 1 (**A**), dataset 2 (**B**), dataset 3 (**C**), dataset 4 (**D**), and dataset 5 (**E**). PDH, postoperative delayed hyponatremia.


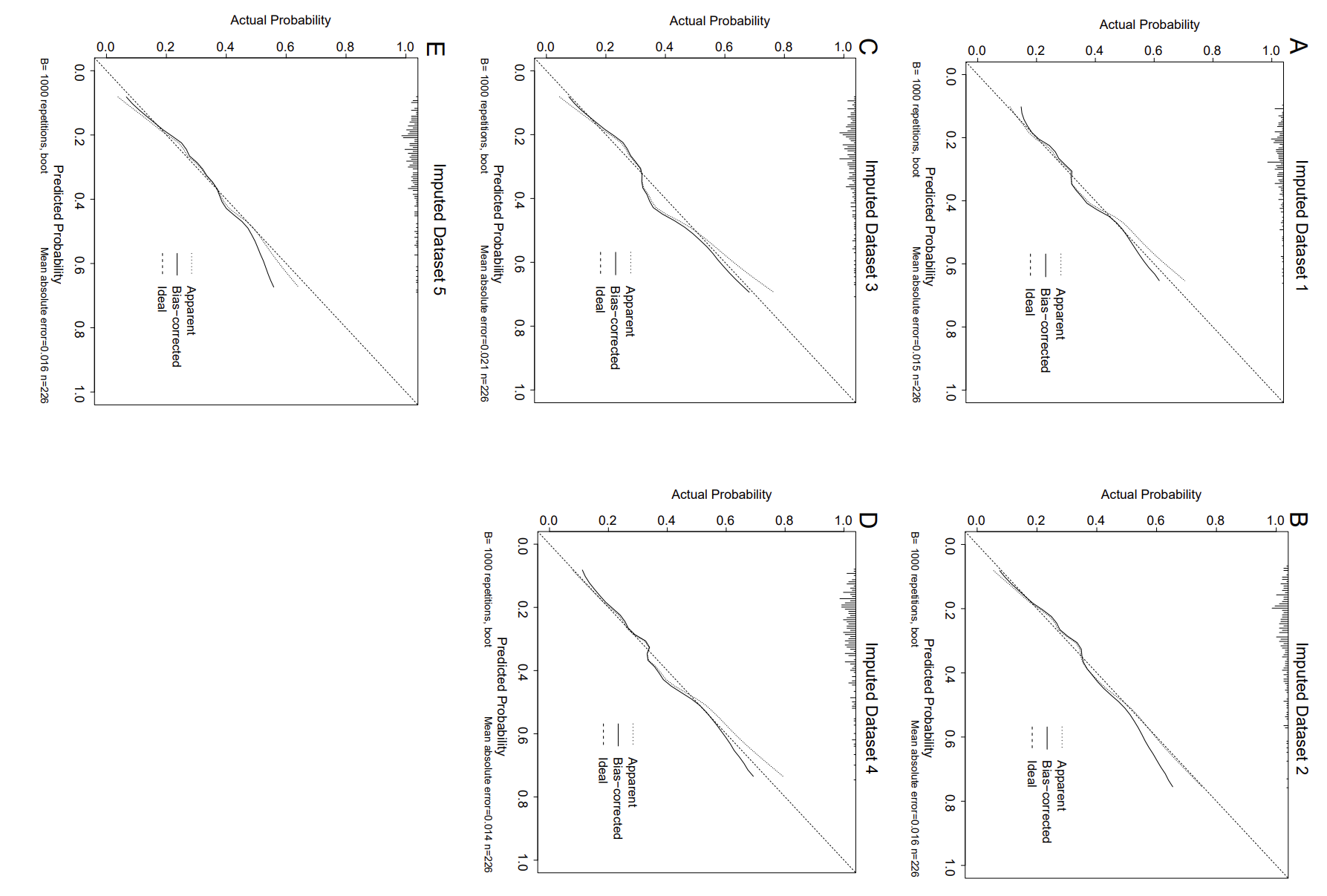


**Supplementary Figure 5.** Decision curve analysis with 95% confidence interval of the nomogram from the imputed dataset 1 (**A**), dataset 2 (**B**), dataset 3 (**C**), dataset 4 (**D**), and dataset 5 (**E**).


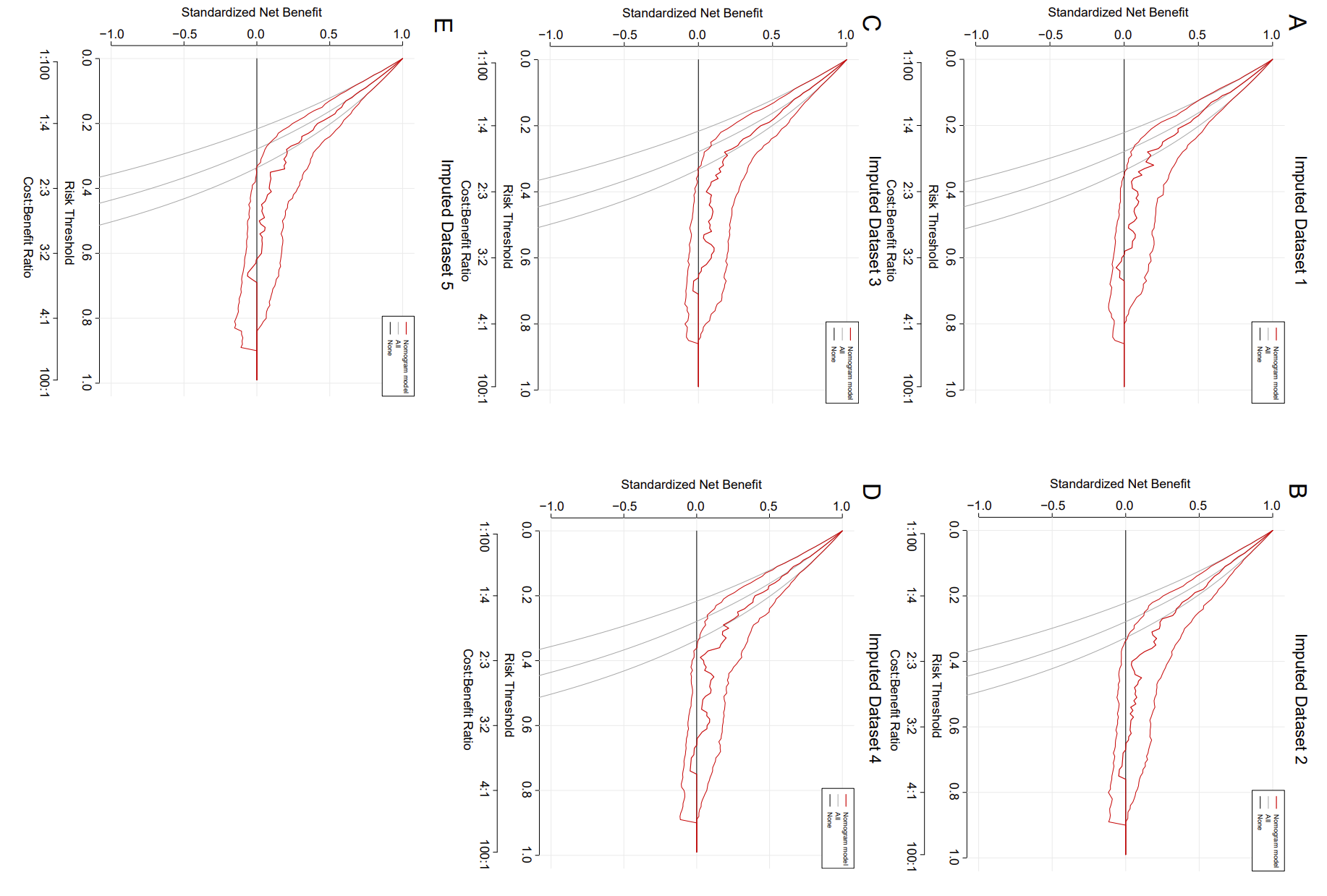

Supplement: Supplementary file 1 — Additional file 1: Supplementary Table 1. TRIPOD checklist for the prediction model development and validation. Supplementary Table 2. Summary of missing data. Supplementary Table 3. Other characteristics of patients in the without PDH group and in the with PDH group. Supplementary Table 4. Univariable logistic regression analysis of the other characteristics. Supplementary Table 5. Spearman correlation analysis between some variables. Supplementary Table 6. Internal and external validation based on AUCs of the nomogram model in the complete dataset and 5 imputed datasets. Supplementary Table 7. Subgroup analysis based on AUCs of the nomogram model in the complete dataset and 5 imputed datasets. Supplementary Figure 1. The missing data patterns. Each row represents a missing pattern. Red and blue blocks indicate missing data and available data, respectively. The left y axis shows the number of missing data in the corresponding pattern. The right y axis shows the number of samples with the corresponding pattern. The bottom x axis shows the number of missing data for each variable. Supplementary Figure 2. The density plots of data before (black line) and after imputation (red line) show good imputation. Supplementary Figure 3. ROC analysis of the nomogram model and variables in the final model from the imputed dataset 1 (A), dataset 2 (B), dataset 3 (C), dataset 4 (D), and dataset 5 (E). AUC, area under the curve. Supplementary Figure 4. Calibration plots of the nomogram from the imputed dataset 1 (A), dataset 2 (B), dataset 3 (C), dataset4 (D), and dataset 5 (E). PDH, postoperative delayed hyponatremia. Supplementary Figure 5. Decision curve analysis with 95% confidence interval of the nomogram from the imputed dataset 1 (A), dataset 2 (B), dataset 3 (C), dataset 4 (D), and dataset 5 (E). [file 41016_2023_334_MOESM1_ESM.docx]
